# Endoscopic discectomy for L4–L5 disc herniation: percutaneous endoscopic transforaminal discectomy vs. unilateral biportal endoscopic discectomy

**DOI:** 10.3389/fsurg.2025.1565165

**Published:** 2025-06-20

**Authors:** Huan Chen, Long Chen, Yu Zhang

**Affiliations:** ^1^ Guangdong Provincial Second Hospital of Traditional Chinese Medicine, Guangzhou, China; ^2^ Guangzhou University of Chinese Medicine, Guangzhou, China

**Keywords:** unilateral biportal endoscopic, L4–L5, lumbar disc herniation, percutaneous endoscopic transforaminal discectomy, unilateral biportal endoscopic discectomy (UBED)

## Abstract

**Study design:**

Retrospective evaluation.

**Objective:**

This study aims to compare the clinical outcomes of percutaneous endoscopic transforaminal discectomy (PETD) and unilateral biportal endoscopic discectomy (UBED) in the management of L4–L5 disc herniation, and to identify the procedure most suitable for different types of herniations.

**Methods:**

Data were retrospectively collected from patients with L4–L5 disc herniation who underwent PETD or UBED between March 2018 and December 2019. Each group consisted of 34 consecutive patients. Key outcomes, including blood loss, operation time, fluoroscopic time, hospitalization duration, and herniation type, were analyzed and compared. Clinical efficacy was assessed using the Oswestry Disability Index (ODI), Visual Analog Scale (VAS), and modified MacNab criteria.

**Results:**

Significant differences were observed between the groups in terms of blood loss, operation time, and fluoroscopic time. Both groups demonstrated significant improvements in VAS scores for back and leg pain, as well as ODI. The proportion of patients achieving excellent or good outcomes was 88.2% for PETD and 91.2% for UBED. Notably, the PETD group had a higher proportion of intervertebral foramen-type disc herniations (32.4%) compared to the UBED group (2.9%; *P* < 0.05). Migration-type herniations were more frequently removed with UBED (35.3%) than with PETD (5.9%; *P* < 0.05).

**Conclusion:**

PETD is associated with less blood loss and shorter operation time, making it the preferred choice for intervertebral foramen-type herniations. UBED, with its shorter fluoroscopic time and reduced puncture difficulty, is more suitable for migration-type herniations. Both techniques are effective for treating central, axillary, and shoulder-type disc herniations. With proper patient selection, both PETD and UBED are safe and effective for L4–L5 disc herniation.

## Introduction

For patients with lumbar disc herniation (LDH) who fail to respond to conservative treatment for more than six weeks, a variety of minimally invasive surgical options are available, such as micro-endoscopy, percutaneous endoscopy and unilateral biportal endoscopy (1). MED is currently considered a standard procedure. Because MED uses a gradual muscle dilatation to establish working cannula, trauma to the paraspinal muscles is reduced (2). However, there is a risk of chronic low back pain and segmental instability after MED (3, 4). From 2006 to 2007, Rutten and chio et al. presented percutaneous endoscopic transforaminal discectomy (PETD) and percutaneous endoscopic interlaminar discectomy (PEID) (5, 6). PETD and PEID used a 6.9 mm single cannula and normal saline medium with satisfactory clinical efficacy and less trauma than MED (4). However, this technique differs from the habits of spine surgeons, and thus the learning curve is steep (7). In recent years, unilateral endoscopic biportal discectomy (UBED) has been introduced by many surgeons. UBED used arthroscopic system and normal saline medium, and obtained satisfactory clinical results (8–10). Unlike PELD, the working and endoscopic portal of UBED are separate. UBED uses a posterior approach, which is consistent with spine surgeons’ habits, so the learning curve is relatively flat (11).

L4–L5 disc herniation presenting with neuronal claudication and radicular radiation pain often severely affects work and learning. Previous literature has reported that PETD is superior to PEID in endoscopic discectomy for L4–L5 disc herniation (12). The relatively large intervertebral foramen in the L4/5 segment provides a good anatomical basis for the use of PETD. On the other hand, most spine surgeons with experience of open surgery are more accustomed to the interlaminar approach, which makes UBED also received a wide welcome. However, it is questionable that the decision of procedure only depends on the surgeon's preferences. Thus far, no study has compared PETD with UBED for L4–L5 disc herniation. The purpose of this retrospective study was to compare the results of PETD and UBED and clarify the selection of appropriate procedure for different patients.

## Methods

This study was approved by the research ethics committee of our institution. The two surgeons are each expert in PETD and UBED. Between March 2018 and December 2019, 34 consecutive patients with L4–L5 LDH received PETD (by Dr. A). 34 consecutive patients received UBED (by Dr. B). The inclusion criteria were unilateral radicular radiation pain and L4–L5 disc herniation which failed to be treated conservatively for 6 weeks. Exclusion criteria were: LDH involving other segments, lumbar spinal stenosis, lumbar spondylolisthesis, fracture, infection, and tumor. According to the location of the disc herniation, it was divided into five types: central, axillary, shoulder, intervertebral foramen and migration. A migratory LDH is defined if the herniated disc is displaced to a greater extent than the height of the posterior edge of the corresponding intervertebral space (13). The blood loss, operation time, fluoroscopic time, hospitalization time, types and complications of the two groups were analyzed and compared. Clinical efficacy was assessed based on the Oswestry Disability Index (ODI), Visual Analog Scale (VAS), and modified MacNab criteria.

### Statistical analysis

All data were presented as mean and standard deviation. Independent samples Mann–Whitney Test was used for comparison intergroup. Intragroup preoperative and postoperative data were compared by paired samples Wilcoxon Signed Ranks Test. All data were analyzed with SPSS software, version 17.0 (SPSS Inc, Chicago, IL) for Windows. Intergroup types and clinical excellence or good rate were compared with a *X*^2^ test. *P* < 0.05 was set as a significant level.

### Surgical techniques

#### PETD

**Figure 1:**
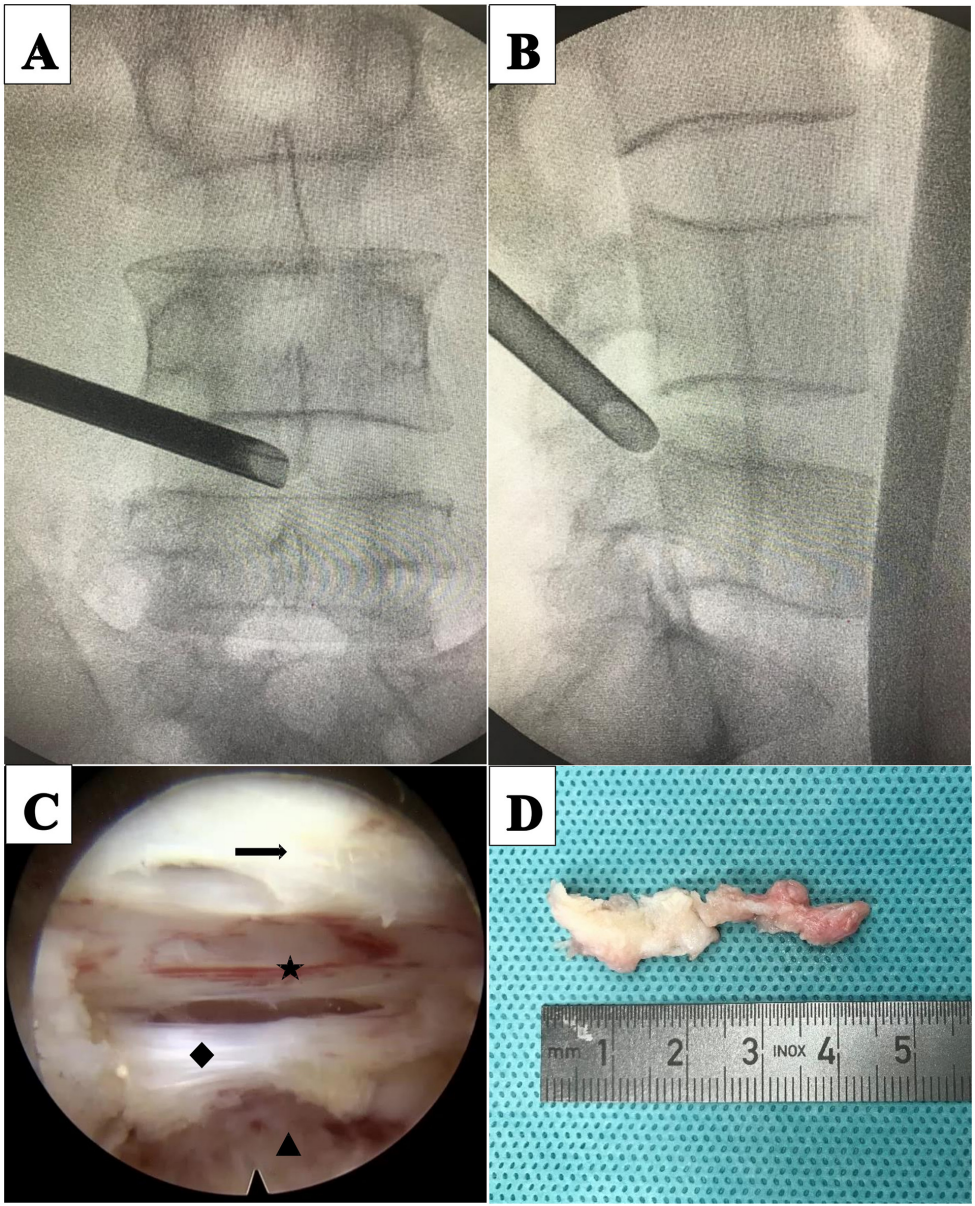
Percutaneous endoscopic transforaminal discectomy (PETD) for a 64-year-old male patient with L4–L5 disc herniation. **(A)** Intraoperative anteroposterior x-ray shows the position of the working cannula. **(B)** Intraoperative lateral x-rays shows the working cannula reaching the ventral side of the dural sac. **(C)** Intraoperative images; (arrow) ligamentum flavum, (star) dural sac, (diamond) posterior longitudinal ligament, (triangle) disc. **(D)** Disc pulposus.

**Figure 2:**
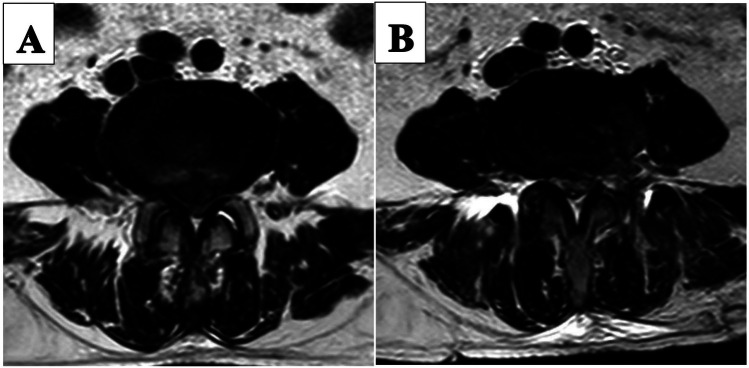
Pre-and post-operative MRI images of a patient in the PETD group. **(A)** Preoperative MRI image shows central disc hermiation. **(B)** 7 days postoperative MRI image.

The patient was placed in prone position on a cushion with genuflex and hip-flexion and the abdomen suspended. The puncture approach was marked on the skin by fluoroscopy. The operation area after disinfection shop waterproof towel. Local anesthesia was performed with 0.5% lidocaine, followed by fentanyl sedation if necessary. A 16-gauge needle punctured the skin 10–14 cm (depending on the patient's size) from the midline of the paravertebral spinous process and through the intervertebral foramen to the target. The needle core was withdrawn and a guide wire was inserted. After the guidewire was fixed at the target, the needle was withdrawn. Extend the skin incision to 8 mm. Series dilators were sequentially inserted to dilate tissue. The primary dilator was placed at the target, while the other dilators were placed on the superior articular process. A protective cannula was placed and the primary dilator was retained and the other dilators were withdrawn. Under fluoroscopic monitoring, trepan was used to perform the foraminaloplasty. During foraminaloplasty, the orientation of the trepan should be adjusted according to the position of the target. The tip of trepan reached the connecting line of the internal edge of the pedicle. The protective cannula and trepan were withdrawn and the working cannula was inserted (Figure 1). The endoscope was then placed within the working cannula. Under continuous irrigation with normal saline, a bipolar flexible radiofrequency probe was used to stop bleeding and provide a clear surgical field of view. Pituitary forceps were used to remove the ruptured annulus fibrosus and the herniated nucleus pulposus (Figure 1). Finally check whether there was any residue left. (Figure 2) shows the preoperative central disc herniation and the postoperative MRI changes at 7 days in a patient from the PETD group.

#### UBED

**Figure 3:**
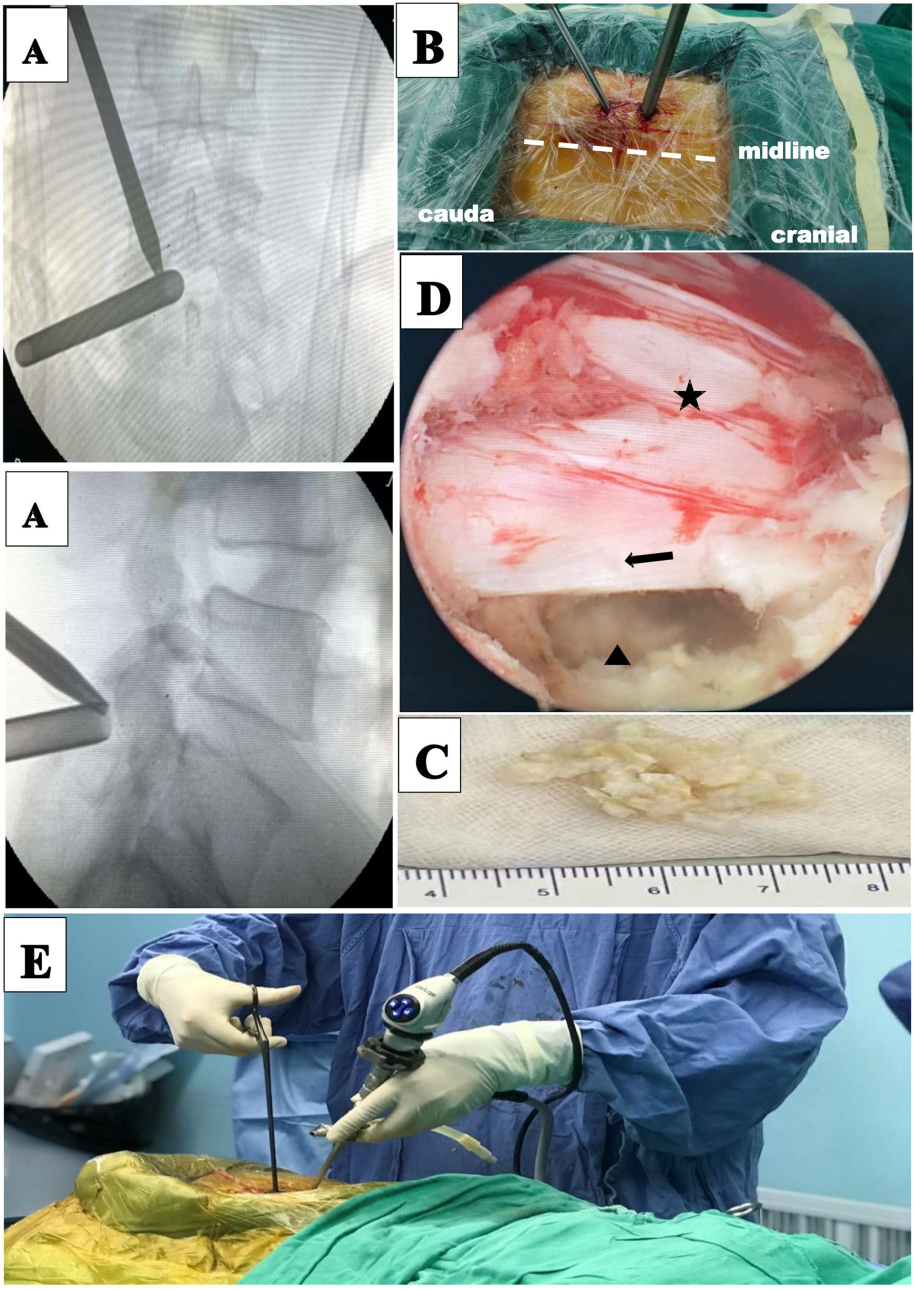
Unilateral biportal endoscopic discectomy (UBED) for a 69-year-old female patient with L4–L5 disc herniation. **(A)** Intraoperative anteroposterior x-ray shows the locations of the two cannulas. **(B)** Portal placements for the UBED. **(C)** Disc pulposus. **(D)** Intraoperative images: (star) dural sac, (arrow) nerve root, (triangle) disc. **(E)** Intraoperative overview of UBED.

**Figure 4:**
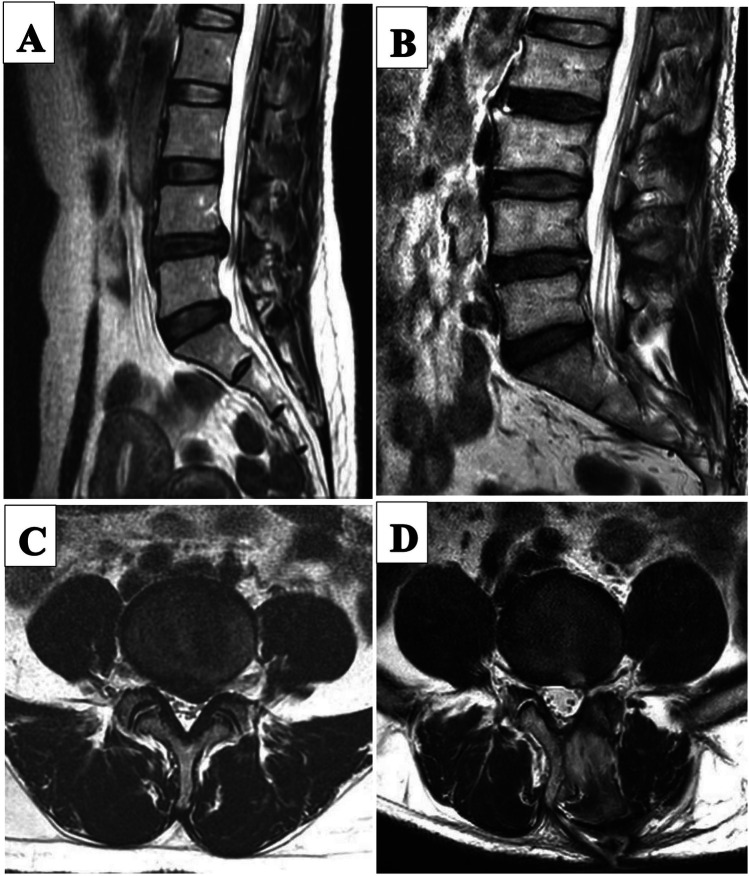
Pre-and post-operative MRI images of a patient in the UBED group. **(A)** Preoperative sagittal MRI image. **(B)** 11 month postoperative sagittal MRI image. **(C)** Preoperative transverse MRI image. **(D)** 11 months postoperative transverse MRI image.

The procedure was performed under general anesthesia. The patient was placed in the prone position and the operating table was adjusted so that the patient's waist was in the forward flexion position. Under fluoroscopy, the biportal incision in the skin was marked. Both incisions were located on either side of the cranial caudal in the superior third plane of the intervertebral space, 1.5 cm from this plane. And the two incisions are positioned on the connecting line of the inner edge of the pedicle. The operation area after disinfection shop waterproof towel. The cranial incision, 6 mm in length, was the access to the endoscope, and the caudal incision, 15 mm in length, was the access to the operation instrument. The endoscopic and the working cannula met at the upper margin of the laminal space (Figure 3). The endoscope was an arthroscope at a 30 angle. Surgical instruments were the same as those used for MED. Normal saline flowed in from the endoscope and overflowed from the working portal. Endoscopic removal of the soft tissue between the paravertebral muscle and the ligamentum flavum was performed using bipolar radiofrequency electrode and pituitary forceps. The ligamentum flavum, upper and lower laminae, and the medial of inferior articular process were exposed. According to the location of the target, a power drill was used to remove part of the bony structure. Fenestration was performed on the ligamentum flavum using pituitary forceps and laminectomy forceps. Epidural adipose tissue was removed to expose the dural sac and nerve root. A clear surgical field of view relied on moderate water pressure and a bipolar flexible radiofrequency probe. The nerve root was protected by the retractor, and the ruptured annulus fibrosus and herniated nucleus pulposus were removed (Figure 3). Figure 4 illustrates preoperative and 11-month postoperative sagittal and axial MRI scans of a patient in the UBED group.

## Results

In the PETD group, the mean age was 53.3 ± 2.9 years, the follow-up time was 14.1 ± 0.4 months, the blood loss was 3.3 ± 2.1 ml, the operation time was 40.0 ± 2.0 min, the fluoroscopic time was 10 ± 0.5 s, and the hospital stay was 2.0 ± 0.3 days. In the UBED group, the average age was 54.9 ± 2.7 years, and the follow-up time was 14.5 ± 0.4 months. The blood loss was 10.1 ± 2.8 ml, the operation time was 57.0 ± 2.0 min, the fluoroscopic time was 5.2 ± 0.2 s, and the hospital stay was 2.1 ± 0.2 days. There was no significant difference in age, sex and hospital stay between the two groups (Table 1). A higher number of patients in the PETD group had intervertebral foramen disc herniation (11 cases, 32.4%) compared with that in the UBED group (1 cases, 2.9%; *P* < 0.05). 12 cases (35.3%) of migration were removed using UBED and 2 case (5.9%) was removed using PETD (*P* < 0.05) (Table 1). A significant difference between groups was demonstrated in terms of blood loss, operation time and fluoroscopic time (Table 2). The mean blood loss was greater in the UBED group than in the PETD group. Mean operation time was longer in the UBED group and mean fluoroscopic time was longer in the PETD group. In the two groups, the mean VAS scores for back and leg pain, as well as the ODI, were significantly improved (Table 3). The excellent and good rates in the PETD group and the UBED group were 88.2% and 91.2%, respectively (Table 3). One case in the PETD group still had pain in the leg postoperative (VAS = 4). An MRI examination revealed 1/3 of the residual disc herniation. Rehabilitation after conservative treatment. In one case of UBED group, a needle-tip-sized breach in the dural sac was found intraoperative, but no repair treatment was performed, and no cerebrospinal fluid leakage occurred postoperative.

**Table 1:** General information of patients in the Two groups.

Characteristics	PETD group	UBED group	*P*
n	34	34	
Age (year)	53.3 ± 2.9	54.9 ± 2.7	0.700
Sex (Male:Female)	19:15	20:14	0.806
Disc type			
Central	9	4	0.217
Axillary	3	9	0.112
Shoulder	9	8	0.799
Intervertebral foramen	11	1	0.003
Migration	2	12	0.007

PETD, percutaneous endoscopic transforaminal discectomy; UBED, unilateral biportal endoscopic discectomy.

**Table 2:** Comparison of blood loss, operation time, fluoroscopy time and hospitalization time between the Two groups.

Group	*n*	Blood loss (ml)	Operation time (min)	Fluoroscopy time (s)	Hospitalization time (d)
PETD Group	34	3.3 ± 2.1*	40.0 ± 2.0*	10 ± 0.5*	2.0 ± 0.3
UBED Group	34	10.1 ± 2.8	57.0 ± 2.0	5.2 ± 0.2	2.1 ± 0.2

PETD, percutaneous endoscopic transforaminal discectomy; UBED, unilateral biportal endoscopic discectomy.

* *P* < 0.05 versus UBED group.

**Table 3:** Clinical comparison between PETD and UBED.

Clinical outcomes	PETD group	UBED group	*P*
Preoperative			
VAS Back	4.1 ± 0.2	4.2 ± 0.2	0.701
VAS Leg	7.0 ± 0.1	7.1 ± 0.2	0.565
ODI (%)	22.4 ± 0.4	21.56 ± 0.5	0.240
Postoperative			
VAS Back (1st day)	1.2 ± 0.2*	1.8 ± 0.2*	0.125
VAS Back (1st month)	0.8 ± 0.1*	0.9 ± 0.1*	0.360
VAS Back (3rd month)	0.5 ± 0.1*	0.7 ± 0.1*	0.324
VAS Back (Last follow up)	0.7 ± 0.1*	0.6 ± 0.1*	0.558
VAS Leg (1st day)	1.1 ± 0.2*	1.2 ± 0.2*	0.555
VAS Leg (1st month)	0.8 ± 0.2*	0.6 ± 0.1*	0.361
VAS Leg (3rd month)	0.6 ± 0.1*	0.9 ± 0.1*	0.099
VAS Leg (Last follow up)	0.8 ± 0.1*	0.7 ± 0.2*	0.333
ODI (%)	60.7 ± 0.7*	59.9 ± 1.0*	0.990
Mac Nab			
Excellence	20	18	
Good	10	13	
Fair	3	3	
Poor	1	0	
Excellence/good rate	88.2%	91.2%	0.690
Follow-up period (month)	14.1 ± 0.4	14.5 ± 0.4	0.378

ODI, oswestry disability index; VAS, visual analog scale; PETD, percutaneous endoscopic transforaminal discectomy; UBED, unilateral biportal endoscopic discectomy.

* *P* < 0.05 versus preoperative.

## Discussion

In recent decades, minimally invasive endoscopic surgery for LDH has been increasingly welcomed by surgeons and patients due to its advantages of small trauma, less bleeding, and rapid recovery (1). MED significantly reduced trauma to the paraspinal muscles and is considered a standard procedure for minimally invasive surgery for LDH (2). In 2007, Rutten et al. presented PETD (5). Unlike MED, PETD uses a transforaminal approach and is mediated by normal saline. PETD had a clear intraoperative field of view, a satisfactory outcome and less trauma than MED, but had the disadvantage of a steep learning curve (14). In 2016, Dong et al. presented unilateral biportal endoscopic (UBE) decompression of the lumbar spinal canal (15). Two portals are the most obvious peculiarities of UBED. UBED applied the interlaminar approach, and the learning curve was flatter than that of PETD (7, 11). Endoscopic discectomy may replace MED as the next gold standard for LDH (16).

In this study, the UBED group revealed more mean blood loss and a longer mean operation time. The results are attributed to the following. First, the L4–L5 segment has a relatively wide intervertebral foramen, and the access of the PETD needle to the intervertebral space is not blocked by the bony structure, which helps to shorten the operation time. Second, the working cannula of PETD reaches the target directly, and no extra-spinal canal tissue resection is required intraoperative. The possibility of bleeding is reduced. Third, the UBED technique requires the creation of an artificial workspace between the lamina and the multifidus muscle. During the process of establishing this working space, part of the paravertebral muscles is stripped off, increasing the blood loss. Fourth, the interlaminar space at the L4–L5 segment is relatively narrow. Part of the laminar and inferior articular process need to be removed, which increases the operation time and intraoperative blood loss. Fifth, the incision size of PETD is 8 mm, while that of UBED is 6 mm and 15 mm, respectively, which is also the reason for the difference in blood loss between the two groups. Anatomical structure, establishment of working portal and incision are the reasons that caused UBED to be inferior to PETD in blood loss and operation time.

The fluoroscopic time of PETD is significantly longer than that of UBE. The reason for this is considered as the distance from the skin puncture site to the target is long for PETD, which dependes more on fluoroscopy. Furthermore, multiple fluoroscopy is required to correct the entry direction and depth of the trepan or drill for PETD in the foraminaloplasty. There was also study that confirm that PETD had more fluoroscopy than UBED and MED, and they believed that the less invasive the surgery, such as PETD, the more fluoroscopy (17).

There was no significant difference in hospital stay and excellent and good rate between PETD group and UBE group. The ODI and VAS scores of the two groups in the early postoperative period and the last follow-up were significantly improved compared with preoperative. Therefore, the results of the two procedures were satisfactory (Figures 2,4). Moreover, trauma to normal lumbar anatomy can be further reduced in comparison with MED (4).

This study had demonstrated that PETD had superiority over UBED in the treatment of intervertebral foramen LDH. When the working portal for PETD was established, it was placed in the intervertebral foramen and the hernia could be easily removed. In UBED, part of the upper lamina and zygapophyseal need to be removed, which increases the operation time and trauma. Treatment with PETD is difficult for the migratory type, especially for the upturn migratory type. The working cannula of PETD has a limited range of motion within the spinal canal and there may be residual hernias. UBE is not limited by bony structures, and fenestration can be performed on the lamina according to the hernia position, thus safely and effectively removing the hernia. Choi et al. studied the efficacy comparison of PETD and PEID for L5-S1 disc herniation. They considered that PETD had advantages in central type and shoulder type, while axillary type and migratory type were more suitable for PEID (18). The PEID and UBED techniques have some similarities, both of which adopt the interlaminar approach. However, this study had shown that PETD was comparable to UBE in central, axillary, and shoulder types. Unlike the S1 nerve root, L5 is separated from the dural sac at the lower half of the L4–L5 intervertebral space, and an axillary hernia easily pushes L5 dorsally. Therefore, the risk of injury to the L5 nerve root by PETD in the removal of axillary type is low. However, in the treatment of shoulder and central type by UBE, due to its wide endoscopic field of view and large movement range of operating instruments, it is easy to pull the nerve root to the midline side, thereby removing the hernia. Dong et al. reported the use of UBED for far lateral disc herniation and bilateral disc herniation (8, 10).

PETD is a better option for patients with a history of posterior approach surgery. The risk of injury to the dural sac using UBE is high in LDH patients with epidural scars (19). The working cannula for PETD is ventral to the dural sac. Resection of the ligamentum flava and the posterior longitudinal ligament is not required, and the pulling amplitude for nerve root is small. Endoscopic surgery using an interlaminar approach can lead to complications such as dural laceration, epidural hematoma, nerve injury and facet fracture (20). In one case of UBED group, a needle-tip-sized breach in the dural sac was found intraoperative, but no repair treatment was performed, and no cerebrospinal fluid leakage occurred postoperative. This case occurred at an early stage of the UBED technique, when the dural sac was accidentally injured while the ligamentum flava was being excised. Kim et al. have shown that a dural sac tear <10 mm can be successfully treated using patch technology (21). There is a risk of injury to the nerve roots and the dural sac because they are inevitably pulled during the posterior approach. In the treatment of L4–L5 disc herniation by UBE, partial laminectomy and medial aspect of inferior articular process are needed, which may lead to lumbar instability (19, 22). However, studies have also revealed that instability occurs only when both laminae and zygapophyseal are removed (8, 23). Preserving the laminar isthmus intraoperative may help to avoid postoperative lumbar instability. PETD adopts local anesthesia, which is suitable for patients with poor general condition who cannot receive general anesthesia.

The effectiveness of UBE in the treatment of LDH has been confirmed by many studies (8, 15, 24). Unlike UBED, single portal endoscopes, such as PETD, with limited operating space and a narrow field of view, especially in the lateral recess, may be difficult to fully expose (25). One case in the PETD group still had pain in the leg postoperative (VAS = 4). An MRI examination revealed 1/3 of the hernia remained. It was considered to be blindness in the exploration of the lateral recess using the nerve hook and there was residual hernia. Nakamura et al. also demonstrated that the greatest weakness of PETD is the residual hernia and inadequate decompression (14). Accurate placement of the working cannula to the target is the most effective way to avoid residual hernia. Zhou et al. suggested that PETD had fewer complications but a steep learning curve due to its minimal trauma to the muscle-ligament complex (26). The UBED only needs the traditional arthroscopy and conventional spinal instruments, and the posterior approach is more in line with the habits of spinal surgeons, which contributes to the extensive development of this technique. Choi et al. revealed that although the learning curve of UBED was relatively flat, the overall incidence of early complications was 10.3% (27). Surgeons during the early stages of developing UBED may encounter problems with obstructed water flow intraoperative. Poor water flow leads to muscle swelling, resulting in a smaller operating space and blurred vision (28). Ahn et al. revealed that the longer the operation time of UBED was, the more obvious the MRI changes of postoperative multifidus muscles would be (29). We believe that the effective establishment of the artificial space between the lamina and multifidus muscle and the identification of anatomical landmarks are the key to improve the efficiency and safety of surgery.

However, this study also has the following limitations. First, this study is a retrospective study, and the sample size is small (*n* = 34 per group), which may result in limited statistical power. Future studies should consider increasing the sample size to enhance statistical power and validate these findings. Second, the non-randomized design and the fact that different surgeons performed the procedures may introduce selection bias, which could affect the generalizability of the results. Future studies should aim to reduce such biases by adopting randomized designs. Third, the retrospective nature of this study limits the ability to make causal inferences, and the results should be interpreted with caution. Future prospective studies with larger sample sizes are needed to further confirm these findings. Additionally, the follow-up time was relatively short, limiting the ability to assess the long-term outcomes of the procedures. Future research should incorporate longer follow-up periods to evaluate the sustained benefits of these procedures. In conclusion, although this study provides valuable initial results, further confirmation through large-scale prospective studies with extended follow-up periods is necessary to validate these outcomes and assess their long-term effectiveness.

## Conclusion

PETD offers several advantages, including reduced blood loss and shorter operation time, making it the preferred approach for treating intervertebral foramen-type disc herniations. UBED, on the other hand, provides benefits such as reduced fluoroscopic time, lower puncture difficulty, a wider field of view, and enhanced flexibility in instrument manipulation, which makes it particularly advantageous for treating migration-type herniations. Both techniques are effective for central, axillary, and shoulder-type lumbar disc herniations. With appropriate patient selection, both PETD and UBED have demonstrated high safety and efficacy in treating L4–L5 disc herniations. However, PETD may be more advantageous for certain herniation types, while UBED appears to offer superior outcomes in cases involving migration-type herniations. Further prospective studies with larger sample sizes and longer follow-up periods are necessary to validate these findings and provide more definitive clinical guidance.

## Data Availability

The datasets presented in this study can be found in online repositories. The names of the repository/repositories and accession number(s) can be found in the article/Supplementary Material.
